# Bone Morphometric Evaluation around Immediately Placed Implants Covered with Porcine-Derived Pericardium Membrane: An Experimental Study in Dogs

**DOI:** 10.1155/2012/279167

**Published:** 2012-11-25

**Authors:** Ryo Jimbo, Charles Marin, Lukasz Witek, Marcelo Suzuki, Nick Tovar, Ioana Chesnoiu-Matei, Irina Florentina Dragan, Paulo G. Coelho

**Affiliations:** ^1^Department of Prosthodontics, Faculty of Odontology, Malmö University, 205 06 Malmö, Sweden; ^2^Department of Dentistry, UNIGRANRIO, Brazil; ^3^Department of Chemical Engineering, Oklahoma State University, Stillwater, OK 74078, USA; ^4^Department of Prosthodontics, Tufts University School of Dental Medicine, Boston, MA 0211, USA; ^5^Departments of Biomaterials & Biomimetics and Periodontology & Implant Dentistry, New York University College of Dentistry, New York, NY 10010, USA; ^6^Department of Periodontology, Tufts University School of Dental Medicine, Boston, MA 0211, USA

## Abstract

*Objective*. To investigate whether porcine-derived bioresorbable pericardium membrane coverage enhances the osseointegration around implants placed in fresh extraction sockets. *Study Design*. Twenty-four commercially available endosseous implants were placed in the fresh extraction sockets of the mandibular first molar of mature beagles (*n* = 6). On one side, implants and osteotomy sites were covered with porcine-derived bioresorbable pericardium membranes, whereas on the other side, no membranes were used. After 6 weeks, samples were retrieved and were histologically processed for histomorphometric analysis. *Results*. The histological observation showed that bone loss and soft tissue migration in the coronal region of the implant were evident for the control group, whereas bone fill was evident up to the neck of the implant for the membrane-covered group. Bone-to-implant contact was significantly higher for the membrane-covered group compared to the control group, 75% and 45% (*P* < 0.02), respectively. *Conclusion*. The experimental membranes proved to regenerate bone around implants placed in fresh extraction sockets without soft tissue intrusion.

## 1. Introduction

The regeneration and healing of bone is a gradual process, and are constantly prone to soft tissue infiltration, particularly in large defects. In order to enhance the healing process, and at the same time, to prevent the migration of unwanted cells, it has been suggested that segregation of the defects via a membrane barrier is effective [1, 2]. Membranes also sustain blood clots in place and allow time for bone forming cells to reconstruct bone unobstructed, which is beneficial for applications such as implant placement in fresh extraction sockets.

The surgical and restorative advantages of placing dental implants in fresh extraction sockets have been discussed clinically by various authors [3–7] with a sufficient number of in vivo studies supporting that successful osseointegration can be achieved in these situations [8–12]. The so-called immediate implant placement is less invasive and potentially more efficient than the classic approach, where multiple surgeries may be needed if using a graft material for the initial bone healing process. However, one of the surgical limitations of an immediate implant placement procedure is that often a socket presents dimensions that may be greater than the diameter of a conventional implant, which at many times results in the presence of a substantial gap between the implant and the socket wall [13] and resorption of the buccal bone wall [14]. It has been proposed that minimizing the gap itself by the use of a conical or a wide diameter implant may be one of the solutions to overcome this problem [15, 16]. However, it has been suggested that full regeneration of the bone is a difficult task, since it seems that alveolar bone resorption to a certain extent is unavoidable regardless of the type of the implant placed [17]. Therefore, the use of a membrane material to create a contained atmosphere could prevent alveolar bone alteration, and simultaneously promote osseointegration. 

Today, a wide variety of membrane materials are commercially available from nonresorbable synthetic to naturally derived membranes. Nonresorbable synthetic membranes, such as polytetrafluoroethylene (PTFE), require removal after 3-4 weeks in order to prevent an immunogenic response. For longer periods, there will be a potential risk of gingival dehiscence, resulting in membrane exposure, moreover infection [18]. On the other hand, naturally derived membranes on the market are mainly manufactured from animal-derived collagen, more specifically, porcine-derived, which are suggested to be biocompatible and biodegradable [19, 20]. This degradation minimizes an immunologic response, possibly reduces patient follow-up visits, and prevents further gingival tissue damage, which has been suggested to be beneficial as compared to the nonresorbable membranes. Studies using the nonresorbable membranes have indeed shown successful bone regeneration due to their excellent space-making and growth factor sustaining properties [21]. However, a recent study showed that even with the resorbable naturally derived membranes, similar biologic outcomes may be expected [22]. These materials are typically fabricated from porcine dermis/peritoneum, or pericardium. Membranes, derived from the porcine peritoneum, are mechanically weaker than their pericardium counterpart [23]. Moreover, since peritoneum-derived membrane barriers naturally present a smooth side and a rough side, the membrane should be oriented in a specific, unidirectional fashion to ensure clinical success. When compared to dermis and other dual-layer membranes, the structure of fibrous pericardium is unique; it has a basement membrane on both sides, resulting not only in a smooth yet porous surface for cellular attachment and proliferation, but also in sufficient density for soft tissue exclusion. 

In this study, the bone morphometry and/or morphology around implants placed in fresh extraction sockets and covered with pericardium derived collagen membrane was evaluated and compared to a group without a membrane, to investigate whether the unique feature of the membrane could provide enhanced bone regeneration.

## 2. Materials and Methods

### 2.1. Surgical Procedures

**Figure 1:**
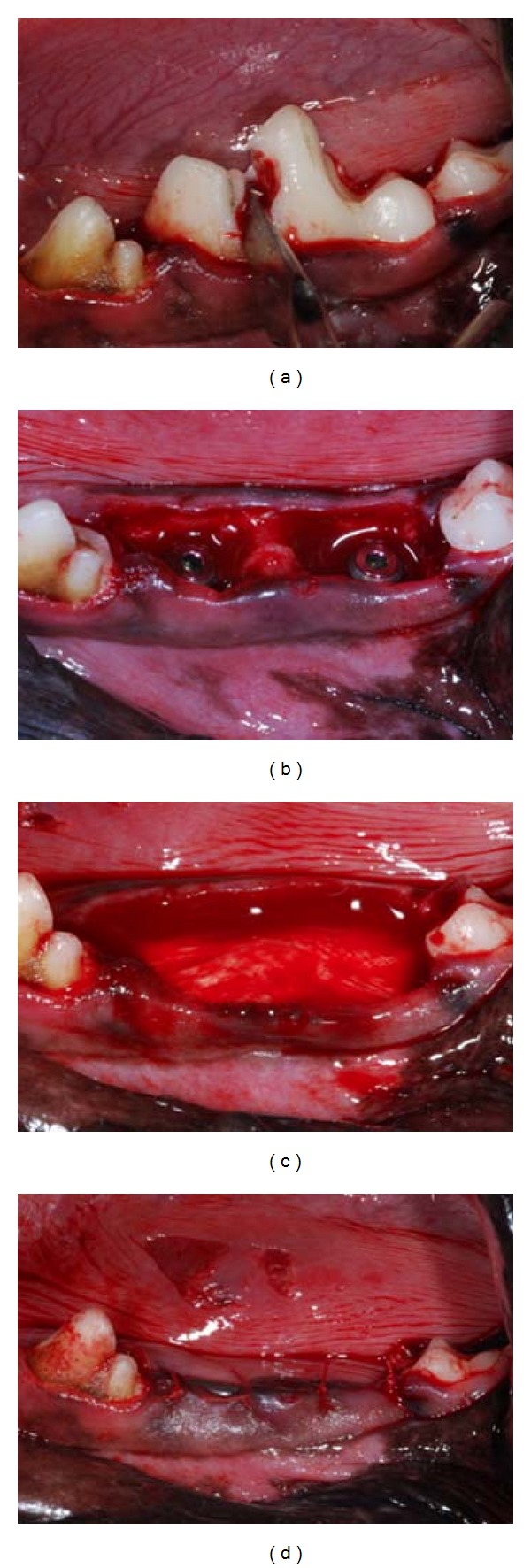
(a) Following sectioning, the teeth were extracted and (b) two 3.3 mm × 13 mm implants were placed in each of the sockets. (c) In one of the sides, a collagen-based membrane was placed and (d) extraction sockets with implants in place were closed with standard suture techniques.

This study used a commercially available pericardium membrane (Vitala, Osteogenics, Lubbock, TX, USA) and 24 commercially available endosseous implants of 3.3 × 13 mm (DT Implants- Ossean Surface, Intra-Lock International, Boca Raton, FL, USA). Following approval of the bioethics committee for animal experimentation (ENVA, France), six beagle dogs with closed growth plates (~1.5 years of age) in good health were acquired for the study and allowed to acclimate for two weeks before surgery. All surgical procedures were performed under general anesthesia. The preanesthetic procedure comprised of an intramuscular administration of atropine sulfate (0.044 mg/kg) and xylazine chlorate (8 mg/kg). General anesthesia was then obtained following an intramuscular injection of ketamine chlorate (15 mg/kg). Bilateral extractions of first mandibular molars were performed (Figure 1(a)). The procedure involved a full thickness mucoperiosteal flap; teeth were sectioned in the buccolingual direction so that individual roots could easily be extracted by root elevators and forceps without any damage to the alveolar bone wall. Following extraction, implants were placed lingually (to replicate the clinical situation) in the mesial and distal sockets, at the buccal bone crest level (Figure 1(b)). Upon measuring with a periodontal probe, it was made sure that a gap of at least 3 mm (range 3.2 to 4.5 mm) was present between the implant body and the buccal side of the alveolar bone. 

On the right side of the mandible, the implants were covered with Vitala (Figure 1(c)); the implants on the contralateral side were used as controls. All implants were placed following the manufacturer's surgical protocol, and primary closure was achieved with resorbable sutures (3-0 Vicryl, Ethicon; Figure 1(d)). Postsurgical medication included antibiotics (penicillin, 20.000 UI/kg) and analgesics (ketoprofen, 1 mL/5 kg) for a period of 48 hours postoperatively. The animals were euthanized 6 weeks after the implant surgery. The euthanasia was performed by anesthesia overdose.

### 2.2. Histological Processing and Quantitative Analysis

Bone block explants consisting of the test and control groups were harvested and processed. The bone blocks were kept in 10% buffered formalin solution for 24 hours, washed in running water for 24 hours, and gradually dehydrated in a series of ethanol solutions ranging from 70% to 100%. Following dehydration, the samples were embedded in a methacrylate-based resin (Technovit 9100; Heraeus Kulzer, Wehrheim, Germany) according to the manufacturer's instructions. The blocks were then cut into slices (300 *μ*m thickness) aiming at the center of the implant along its long axis with a precision diamond saw (Isomet 2000; Buehler, Lake Bluff, IL), glued to acrylic plates with an acrylate-based cement, and a 24-hour setting time was allowed before grinding and polishing. The sections were then reduced to a final thickness of ~30 *μ*m by means of a series of SiC abrasive papers (400, 600, 800, 1200, and 2400; Buehler) in a grinding/polishing machine (Metaserv 3000, Buehler) under water irrigation. The sections were then toluidine blue stained and referred for optical microscopy evaluation. The histologic features were qualitatively evaluated at 50x to 200x magnifications (Leica DM2500M; Leica Microsystems, Wetzlar, Germany). The amount of bone-to-implant contact (BIC) and buccal bone loss (BBL) were calculated by means of a computer software (Leica Application Suite, Leica Microsystems GmbH, Wetzlar, Germany). The regions of bone-to-implant contact along the implant perimeter were subtracted from the total implant perimeter, and calculations were performed to determine the BIC. Wilcoxon matched-pairs test at 95% was utilized for statistical evaluation.

## 3. Results

### 3.1. Histological and Histomorphometrical Evaluation

The surgical procedures and followup demonstrated no complications or other immediate clinical concerns. There were no signs of infection or inflammation at the surgical sites or surrounding tissues throughout the duration of the study.

**Figure 2:**
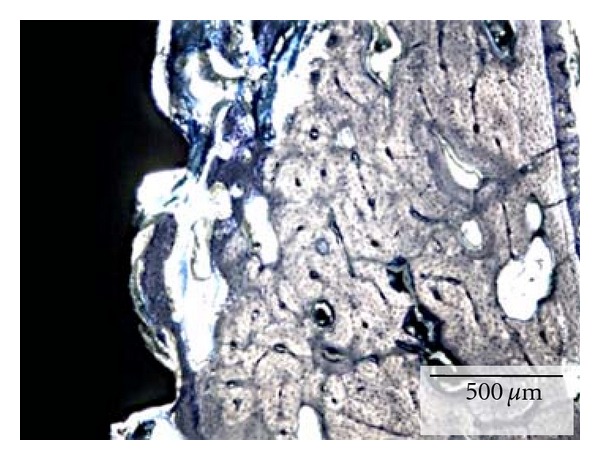
Optical micrograph depicting the new bone filling the gap between the extraction socket wall and implant surface, a common finding for both experimental and control groups.

**Figure 3:**
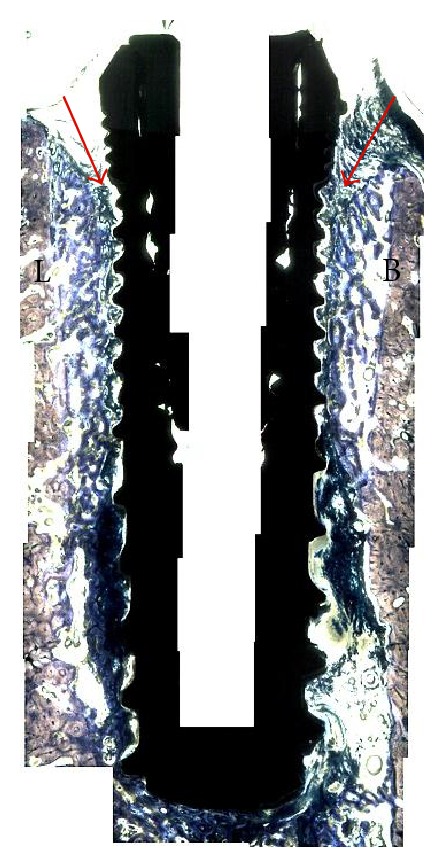
Merged optical micrograph depicting the control group, where the immediate implant placed at the mesial molar socket was not covered with a resorbable membrane prior to suturing. For this group, apical migration (arrows) occurred at both buccal (B) and lingual (L) aspects resulting in lower bone insertion levels along the length of the implant.

**Figure 4:**
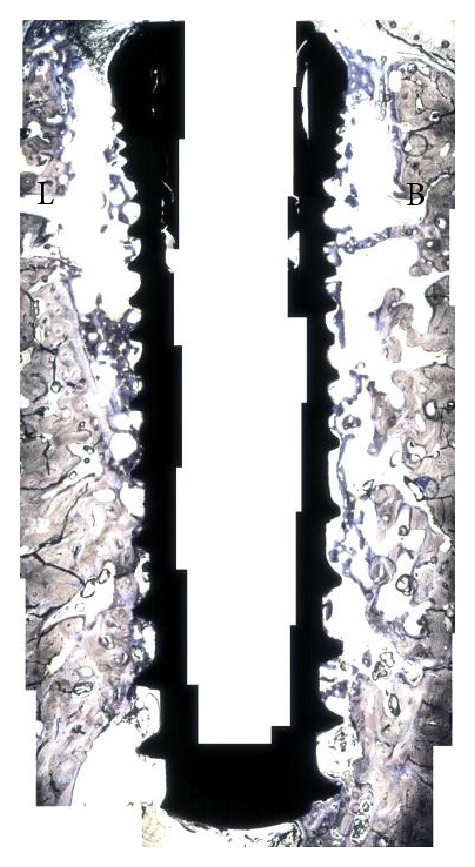
Merged optical micrograph depicting the experimental group, where the immediate implant placed at the mesial molar socket was covered with a resorbable membrane prior to suturing. For this group, extensive apical migration (arrows) did not occur at both buccal (B) and lingual (L) aspects resulting in osseointegration at higher levels relative to the control side. The red and blue boxes are described in detail in Figure 5.

**Figure 5:**
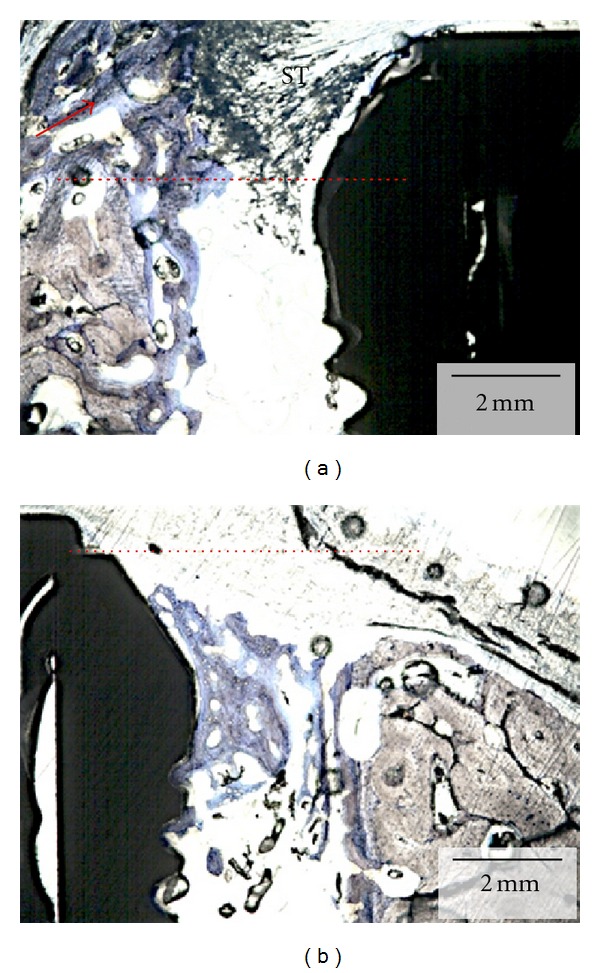
(a) Optical micrograph depicting the lingual aspect of the section presented in the red box in Figure 3. The dashed red line represents the implant cervical level (immediately below the implant cover screw). New bone formation through the course of six weeks resulted in higher levels than at the time of placement (red arrow). The soft tissue (ST) limited migration likely occurred due to membrane movement during suture where it partially bent in the apical direction allowing tissue migration. (b) Optical micrograph depicting the buccal aspect of the section presented in the blue box in Figure 4. The dashed red line represents the implant cervical level (immediately below the implant cover screw). New bone formation through the course of six weeks resulted in bone height maintenance and closure of the gap between implant surface and socket wall. For this section, no soft tissue apical migration occurred.

At 6 weeks, the qualitative analysis of the histologic sections showed for both groups, regions of direct bone-to-implant contact and new woven bone bridging the gap between implant and the old bone of the socket walls (Figure 2). Apical migration of soft tissue resulting in lower cervical to apical bone to implant first contact was observed for the implants in the control group (Figure 3). In the experimental group, where the implants were covered by the membrane at the time of placement, an intimate contact between implant and bone was observed throughout the implant level (Figure 4). Higher magnification optical micrographs of an experimental group section are presented in Figure 5. 

**Figure 6:**
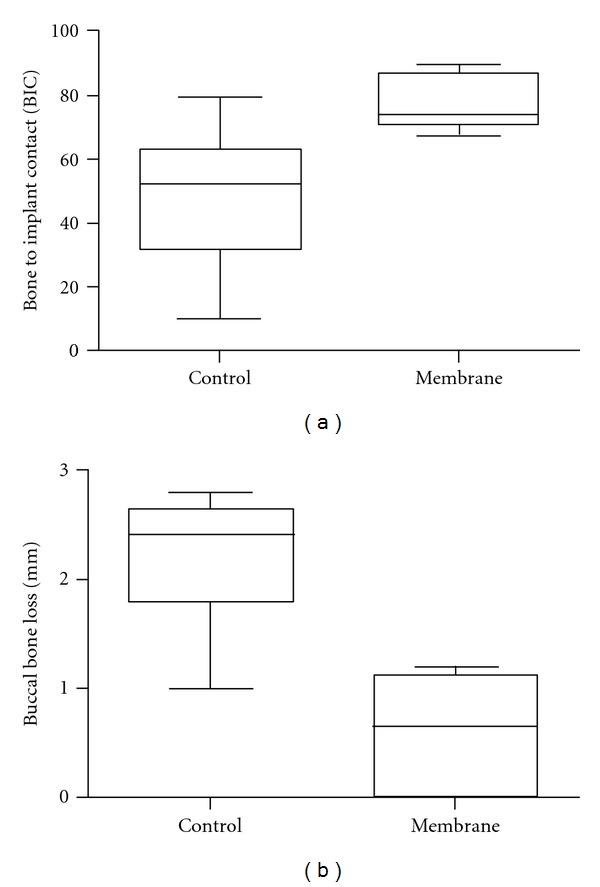
Wilcoxon matched paired test revealed (a) significant higher BIC to the experimental group (*P* < 0.02), and (b) significantly lower buccal bone loss for the membrane group compared to the control group (*P* < 0.02).

Quantitative analysis of the test group rendered a significantly higher BIC in comparison to the BIC observed for the control group, 75% versus 45% (*P* < 0.02), respectively. The sites that were covered with the membrane presented a 0.7 mm buccal bone loss which was significantly lower than the control group that showed a 2.5 mm loss in buccal plate (*P* < 0.02, Figure 6). The implantation site within arch (mesial or distal) did not influence BIC or BBL in either control or test groups (*P* > 0.80). 

## 4. Discussion

Immediate implant therapy has been proven to be a successful clinical treatment, since it is less invasive and is beneficial in shortening the treatment period [24, 25]. The survival rate of immediately placed and loaded implants over a 7–10 year followup varies between 85 and 91%, depending on location of the implant [26]. Following tooth extraction, a discrepancy between the diameter of the extraction socket and an immediate implant renders a gap that can influence the osseointegration of the implant by allowing apical soft tissue migration. In this study, the implants placed in the fresh extraction sockets of dogs showed appropriate osseointegration with direct bone-implant-contact when an occlusive collagen pericardial membrane barrier was used. 

Previous studies have shown that the gap width was one of the decisive factors in order to achieve implant osseointegration [27]; for instance, a gap ranging 0.35–1 mm was shown to result in incomplete bone healing around the implants [28, 29]. Knox et al. evaluated the coronal positioning of the bone-to-implant contact in dogs, in gaps up to 2 mm without the placement of a membrane [30]. Their results showed that the level of coronal bone position along the implant surface was dependent on the initial gap between alveolar bone and implant. These conclusions are supported by the results obtained from the current study where, by eliminating apical soft tissue migration over the implant by means of a membrane barrier, higher levels of BIC, lower buccal bone loss, and a more coronal direct bone apposition was observed compared to controls. 

Although implant surface and implant design play an important role in the osseointegration and survival of implants in fully healed bone [31], the data may not be fully applicable in sites such as implant placement in extraction sockets or in immediate loading conditions [25]. Under progressive and dynamic ridge alteration, the effect of the state of the art surface architecture may be less effective, and the biological reassembly takes initiative in the configuration procedure [14]. Thus, in order to successfully obtain bone fill and osseointegration in a gap created during implant placement in fresh extraction sockets, additional regenerative procedures such as the guided soft tissue/bone regeneration (GTR/GBR) with the use of occlusive membranes may be necessary to maintain space for blood clot infiltration and maturation, further to exclude soft tissue invasion [32].

The appropriate bone regenerative outcomes around the implants placed in fresh extraction sockets of the current study has indicated that the use of membrane to cover the gap is an effective procedure. This is in agreement with other studies using a commercially available bioresorbable membrane, which presented that the use of such membrane contributed to the preservation of the buccal outline of the alveolar process [33, 34]. Moreover, the structural characteristics of the pericardium-derived porcine membrane may have been responsible for further bone enhancing effects since our histologic sections did not show extensive membrane collapsing into the gap between the socket wall and implant bulk. Mechanical property wise, it has been reported that these membranes possess a better tensile strength and ball burst than other collagen membranes derived from small intestine submucosa (peritoneum) or acellular dermal matrix [35]. With regards to the structure of the membrane, the noncross linked matrix derived from the porcine pericardium has a bionic feature [36], which has been suggested to be a key factor for cell migration and morphogenesis [37]. 

To conclude, the findings of the present study showed that using a bioresorbable, pericardium membrane resulted in significantly higher BIC and a closer fit between the bone margin and the abutment-fixture margin as compared to sites without membrane coverage. Although it has not been compared to other membrane materials in the present study, the outcomes of this study strongly suggests the bioeffectiveness of the biologically inspired design membrane in challenging cases such as implant placement in the fresh extraction sockets. Another aspect to further clarify the effect of the membrane is to identify the time course changes in relation to the anatomical landmarks as presented in numerous studies conducted by Araújo et al. [38–40]. In order to bring in clinical benefits, further investigations comparing the pericardium membrane to other membrane materials which are clinically used are necessary.
